# Root-associated bacterial communities and root metabolite composition are linked to nitrogen use efficiency in sorghum

**DOI:** 10.1128/msystems.01190-23

**Published:** 2023-12-22

**Authors:** Yen Ning Chai, Yunhui Qi, Emily Goren, Dawn Chiniquy, Amy M. Sheflin, Susannah G. Tringe, Jessica E. Prenni, Peng Liu, Daniel P. Schachtman

**Affiliations:** 1 Department of Agronomy and Horticulture, Center for Plant Science Innovation, University of Nebraska-Lincoln, Lincoln, Nebraska, USA; 2 Department of Statistics, Iowa State University, Ames, Iowa, USA; 3 Environmental Genomics and System Biology, Lawrence Berkeley National Laboratory, Berkeley, California, USA; 4 Department of Energy Joint Genome Institute, Lawrence Berkeley National Laboratory, Berkeley, California, USA; 5 Department of Horticulture and Landscape Architecture, Colorado State University, Colorado State University, Fort Collins, Colorado, USA; CNRS Delegation Alpes, Lyon, Rhône-Alpes, France

**Keywords:** sorghum, root metabolites, bacterial communities, nitrogen use efficiency, nitrogen stress, biomass

## Abstract

The development of cereal crops with high nitrogen use efficiency (NUE) is a priority for worldwide agriculture. In addition to conventional plant breeding and genetic engineering, the use of the plant microbiome offers another approach to improving crop NUE. To gain insight into the bacterial communities associated with sorghum lines that differ in NUE, a field experiment was designed comparing 24 diverse *Sorghum bicolor* lines under sufficient and deficient nitrogen (N). Amplicon sequencing and untargeted gas chromatography–mass spectrometry were used to characterize the bacterial communities and the root metabolome associated with sorghum genotypes varying in sensitivity to low N. We demonstrated that N stress and sorghum type (energy, sweet, and grain sorghum) significantly impacted the root-associated bacterial communities and root metabolite composition of sorghum. We found a positive correlation between sorghum NUE and bacterial richness and diversity in the rhizosphere. The greater alpha diversity in high NUE lines was associated with the decreased abundance of a dominant bacterial taxon, *Pseudomonas*. Multiple strong correlations were detected between root metabolites and rhizosphere bacterial communities in response to low N stress. This indicates that the shift in the sorghum microbiome due to low N is associated with the root metabolites of the host plant. Taken together, our findings suggest that host genetic regulation of root metabolites plays a role in defining the root-associated microbiome of sorghum genotypes differing in NUE and tolerance to low N stress.

**IMPORTANCE:**

The development of crops that are more nitrogen use-efficient (NUE) is critical for the future of the enhanced sustainability of agriculture worldwide. This objective has been pursued mainly through plant breeding and plant molecular engineering, but these approaches have had only limited success. Therefore, a different strategy that leverages soil microbes needs to be fully explored because it is known that soil microbes improve plant growth through multiple mechanisms. To design approaches that use the soil microbiome to increase NUE, it will first be essential to understand the relationship among soil microbes, root metabolites, and crop productivity. Using this approach, we demonstrated that certain key metabolites and specific microbes are associated with high and low sorghum NUE in a field study. This important information provides a new path forward for developing crop genotypes that have increased NUE through the positive contribution of soil microbes.

## INTRODUCTION

The role of root metabolites in modulating the root-associated microbiome has gained significant attention in recent years. Although it is known that certain plant metabolites exuded from plant roots can shape the composition of the rhizosphere microbiome (1–4), the effects of a substantial number of primary and secondary root metabolites on the root-associated microbiome remain unclear. Root metabolites are likely to influence the microbes that inhabit both the inside of the roots (endosphere) and in the region just outside the roots (rhizosphere). Few studies have characterized the association between the root metabolites and the root-associated microbiome (3, 5, 6), and even fewer studies have investigated how this association is affected by abiotic stresses (7, 8). Since root metabolites are sensitive to changes in environmental conditions (9–11), the study of root metabolites may provide valuable insights into the factors underlying host plant modulation of root-associated microbiomes in response to abiotic stresses. Additionally, the host plant species and genotype are likely to play a crucial role in the interaction between root exudates and the root-associated microbiome. Host plants have been shown to have a significant effect on the composition of the root-associated microbiome (6, 12–15); therefore, understanding the host plant control over the root-associated microbiome is of great interest to crop breeders for the selection of cultivars that are more productive and resilient to stresses.

Over the past few decades, the application of nitrogen (N) fertilizer has intensified to accommodate increased demands for higher crop yields due to rapidly growing populations. While an excess amount of applied N ensures higher yields, it also leads to adverse environmental impacts due to the leaching of N fertilizer into waterways and groundwater. Therefore, to mitigate adverse environmental effects, it will be necessary to lower the input of N fertilizer while selecting crop cultivars with high N use efficiency. *Sorghum bicolor* is an excellent model system for studying this because it requires less N fertilizer than maize, which has been extensively studied by plant biologists and agronomists (16). It also exhibits a range of nitrogen use efficiency (NUE), which may be in part due to the vast genetic diversity within the sorghum gene pool. While past studies have identified genes (17, 18) and phenotypes (19, 20) that are potentially associated with high NUE in sorghum, there is still a lack of information on the overall variation in sorghum NUE and the relationship between NUE and recruitment of root-associated microbes.

As an ancient African grass, elite sorghum lines that are grown as a staple crop worldwide are the result of many generations of breeding concomitant with the selection across geographic gradients (21). Sorghum can be classified into four major types based on their carbon partitioning characteristics (21). Grain sorghum, the most widely grown sorghum type, is primarily grown for food and partitions carbon into panicles. The forage cultivars have lower grain yield, exhibit coarser stems (compared with grain sorghum), and are used mainly for grazing and silage. In contrast, energy and sweet sorghum mostly partition carbon to the stem but in the form of lignocellulosic biomass and fermentable sugars, respectively, making them excellent feedstocks for biofuel production (22, 23). Information about the NUE of energy sorghum will be particularly important for the identification of lines that grow well in marginal soils so this lignocellulosic biomass crop can be grown efficiently with little added N and where it will not compete with food crops.

In this study, we characterized the root metabolite and bacterial communities across 24 diverse sorghum genotypes grown under full and low N field conditions. Our goal was to construct a more comprehensive picture that advances the understanding of the interplay between the host regulation of root metabolites and the root-associated microbiome of sorghum genotypes differing in response to N stress. To achieve this goal, we sought to answer the following questions. (i) How does N stress and sorghum genotype affect the associated bacterial community diversity and composition? (ii) How does N stress and sorghum genotype affect the root metabolite composition? (iii) Are the bacterial communities different between high and low NUE genotypes? (iv) Is there an association between root metabolites and rhizosphere bacterial community composition? This is vital foundational information for our overall understanding of the relationship between root metabolites and associated microbes and could be used in future work to intentionally promote specific plant–microbe interactions that would be more favorable to low N conditions for sorghum and other crop species.

## RESULTS

### A diverse panel of sorghum genotypes exhibits variation in N use efficiency

**Fig 1:**
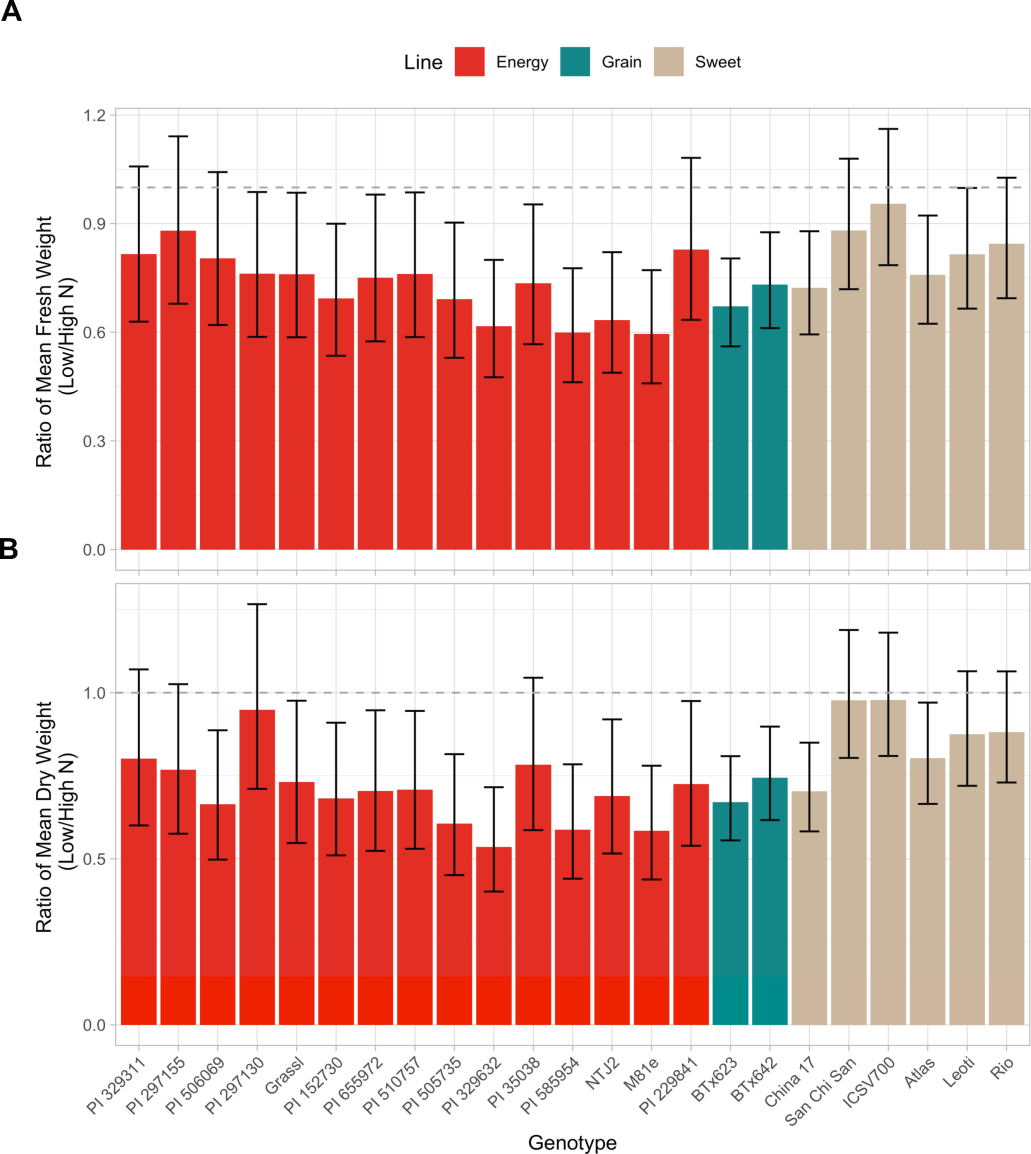
Biomass ratios of 23 diverse sorghum genotypes. (**A**) Fresh and (**B**) dry weight ratios derived by dividing the biomass measured under low *vs* full N conditions. Error bars represent the 95% confidence intervals. *n* = 8 for each genotype. Dashed line represents biomass ratio = 1.

In this study, the NUE of 23 diverse sorghum genotypes that span three sorghum types was compared (Fig. 1; Fig. S1). The sorghum lines were grown under full and low N conditions in the same field site, and their NUE was derived as the ratio of the aboveground biomass under low versus full N conditions measured at the end of the season. The low N treatment significantly reduced the biomass of all the sorghum genotypes (ANOVA: *P* < 0.001). The average reduction in dry biomass due to low N was 22% with some genotypes exhibiting larger reductions in biomass than others. PI 329632 was the most susceptible to low N, exhibiting 40% reduction in dry biomass in response to low N. ICSV700 was the least susceptible to low N as its biomass barely decreased due to low N. The sweet sorghum genotypes used in this study exhibited an average of 23% higher dry biomass ratio than the other two sorghum types, indicating higher NUE in this sorghum type.

### Sorghum rhizosphere and root endosphere microbiome are affected by N availability and sorghum type

**Fig 2:**
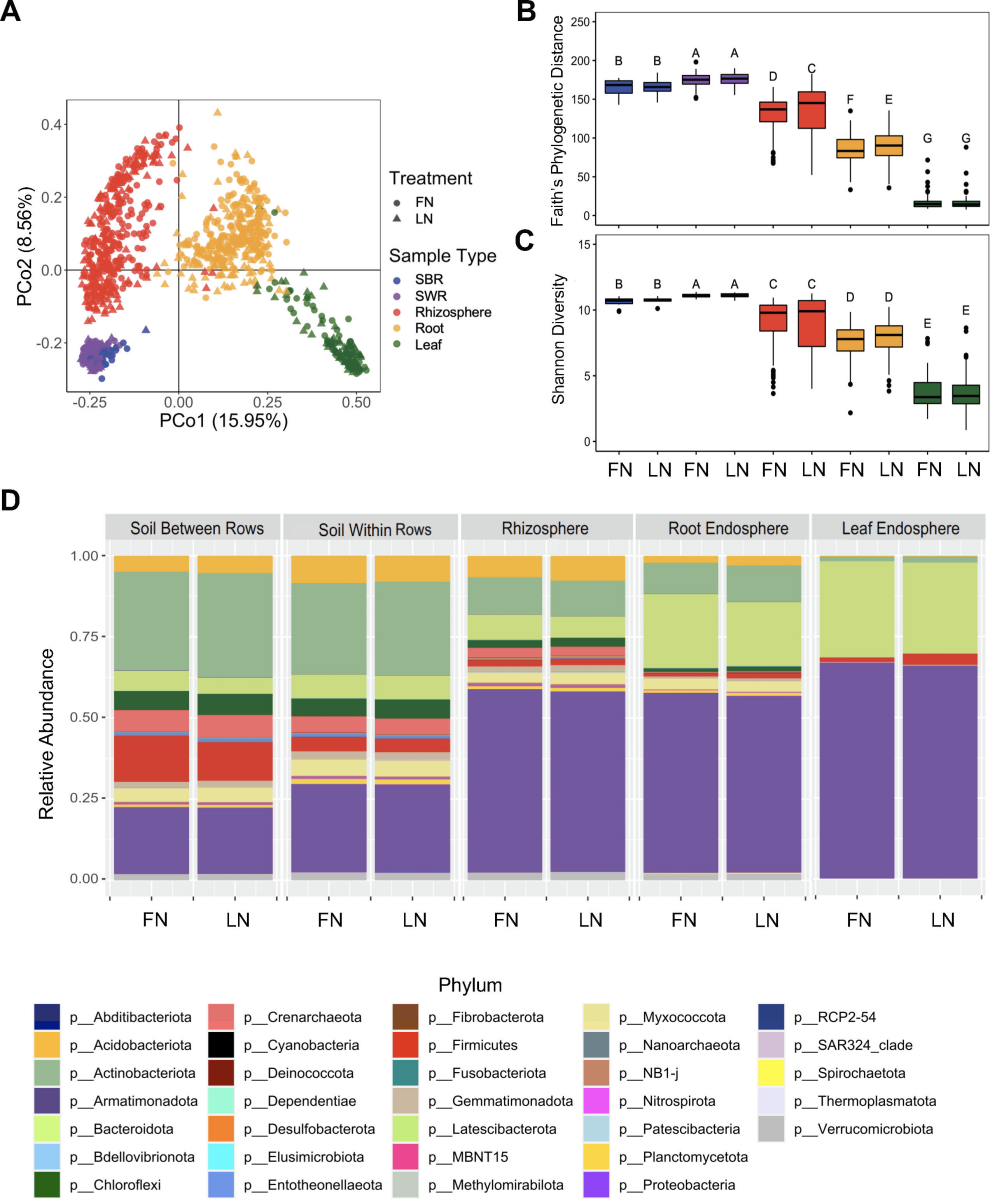
Bacterial diversity and composition across different compartments. (**A**) Principal coordinate analysis (PCoA) based on Bray–Curtis distance showing the overall composition of bacterial communities across different compartments and N treatments. (**B**) Faith’s phylogenetic distance and (**C**) Shannon diversity of bacterial communities for each compartment and treatment combination. (**D**) Relative abundance of the most abundant phyla across different compartments and N treatments. Abbreviation: SWR, soil within rows; SBR, soil between rows; FN, full N; LN, low N.

The bacterial communities in the soil between rows, soil within rows (the soil layer beyond rhizosphere but near roots), rhizosphere, root endosphere, and leaf endosphere at the sorghum vegetative stage were surveyed by sequencing the V4 region of the 16S rRNA amplicons. We found that sample type or compartment (rhizosphere, root and leaf endosphere, and soils) were the major factors that affected the assembly of sorghum microbiomes (Fig. 2A), accounting for 27% of the variation in the microbial communities. The microbial diversity and richness decreased from the soil within rows to the endophytic compartments, with the leaf endosphere harboring the least diverse microbial community (Fig. 2B and C). We also detected a significant increase in bacterial richness in the rhizosphere and root endosphere due to low N (Fig. 2B). From the soil between rows to the endophytic compartments, we observed a substantial change in the relative abundance of the members of various phyla. Notably, the phyla Crenarchaeota, Chloroflexi, Actinobacteriota, and Firmicutes decreased by 95%, 86%, 76%, and 43%, respectively (Fig. 2D). In contrast, an increase of 217% in relative abundance for the phylum of Bacteroidota and 138% for Proteobacteria was measured.

**Fig 3:**
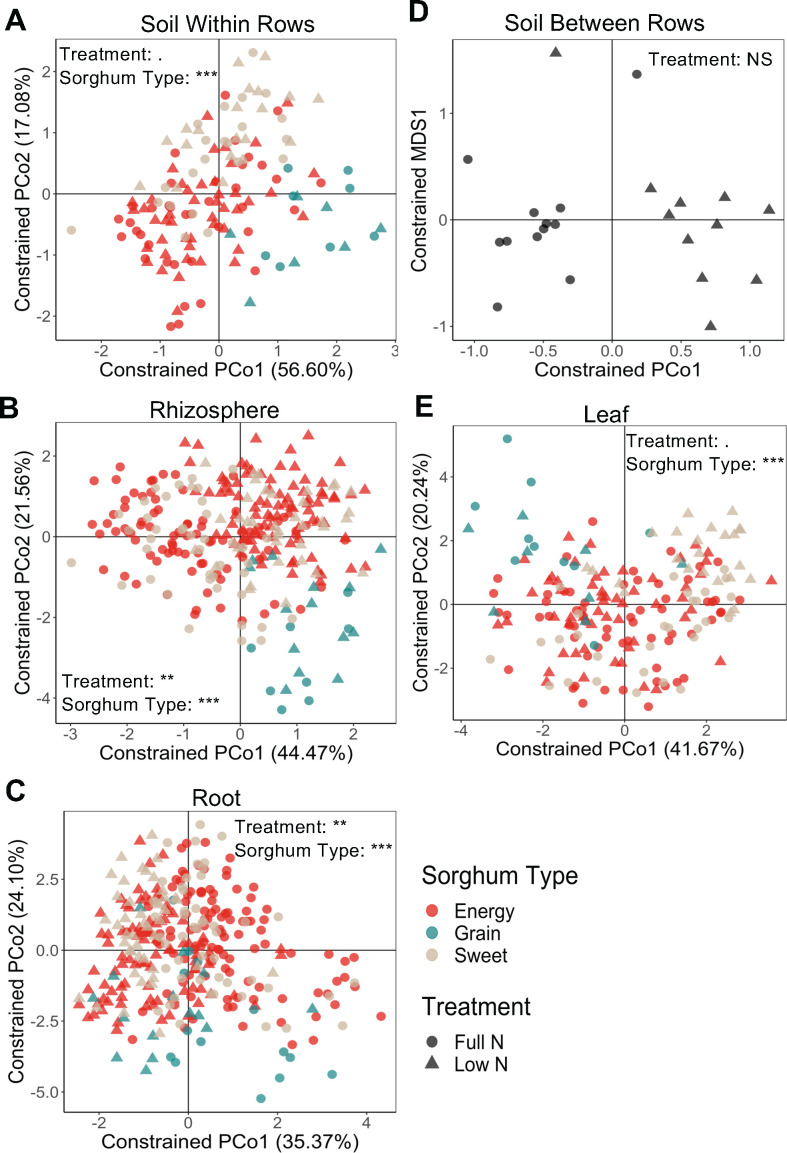
Effects of N treatment and sorghum type on the bacterial community in each compartment. CAP based on Bray–Curtis distance of (**A**) soil within rows, (**B**) rhizosphere, (**C**) root endosphere, (**D**) soil between rows, and (**E**) leaf endosphere. ^.^*P* ≤ 0.1, ^*^P ≤ 0.05, ^**^P ≤ 0.01, and ^***^P ≤ 0.001. Abbreviation: NS, not significant.

**Fig 4:**
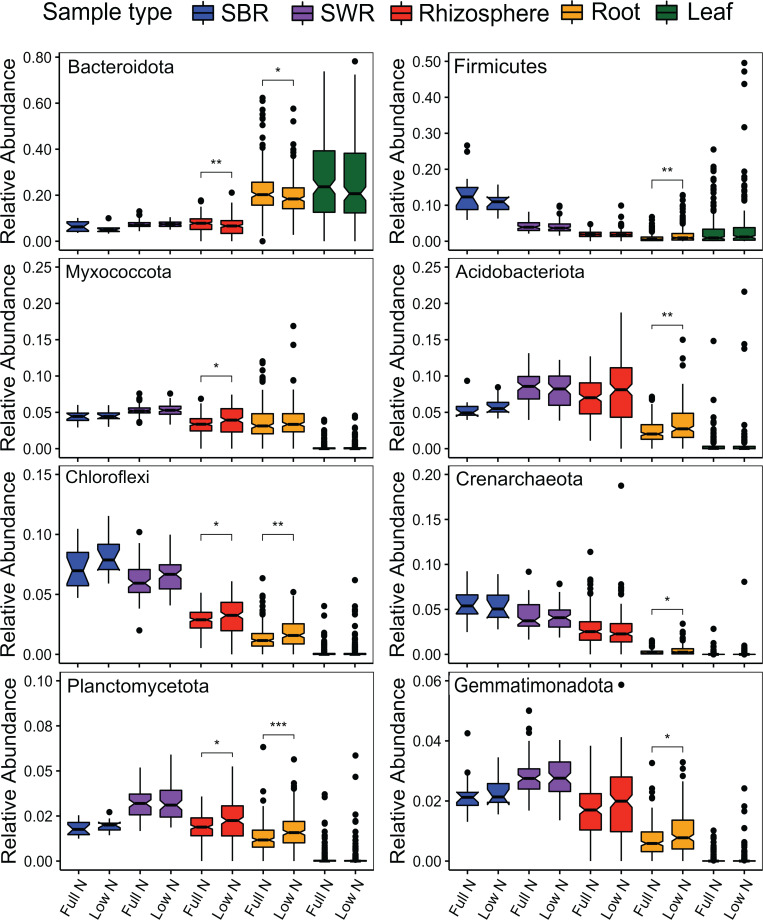
Relative abundance of bacterial phyla that were significantly impacted by N stress in at least one compartment determined by LEfSE. ^*^P ≤ 0.05, ^**^P ≤ 0.01, and ^***^P ≤ 0.001.

Next, we dissected the effects of N availability and sorghum type on the bacterial communities for each compartment (Fig. 3A through E). Canonical analysis of principal coordinates (CAP) and permutational multivariate analysis of variance (PERMANOVA) revealed that the rhizosphere and root endosphere communities were significantly impacted by N availability, which separated the samples along the primary axis (Fig. 3B and C), suggesting that it was a major factor that shaped the microbial communities in these compartments. Linear discriminant analysis effect size (LEfSE) was then used to identify the microbial taxa across all taxonomic ranks that were differentially impacted by N availability for each compartment. Consistent with the CAP and PERMANOVA findings, significant differential abundance at the phylum level was only detectable in the rhizosphere and root endosphere (Fig. 4). Bacteroidota was the only phylum that was significantly enriched under full N conditions, and this was observed for both rhizosphere and root endosphere. Chloroflexi and Planctomycetota were in greater abundance under low N conditions in both rhizosphere and endosphere, while Myxococcota was only enriched in low N rhizosphere samples. The phyla that were significantly enriched under low N conditions in the root endosphere were Firmicutes, Acidobacteria, Crenarchaeota, and Gemmatimonadota. In addition to N availability, we observed a significant effect from the sorghum type (energy, grain, and sweet) in all compartments except for soil between rows, which was the only compartment that was not associated with plants (Fig. 3A through E). Subsequent pairwise PERMANOVA revealed that the microbial communities in soil, root, and leaf were significantly different across the three sorghum types, except in the rhizosphere, where no discernible difference between energy and sweet sorghum was observed (Table S2). A higher similarity between the sweet and energy types compared with grain sorghum was observed across all sample types, regardless of the significance.

### The alpha diversity and composition of the rhizosphere bacterial community are associated with sorghum NUE

**Fig 5:**
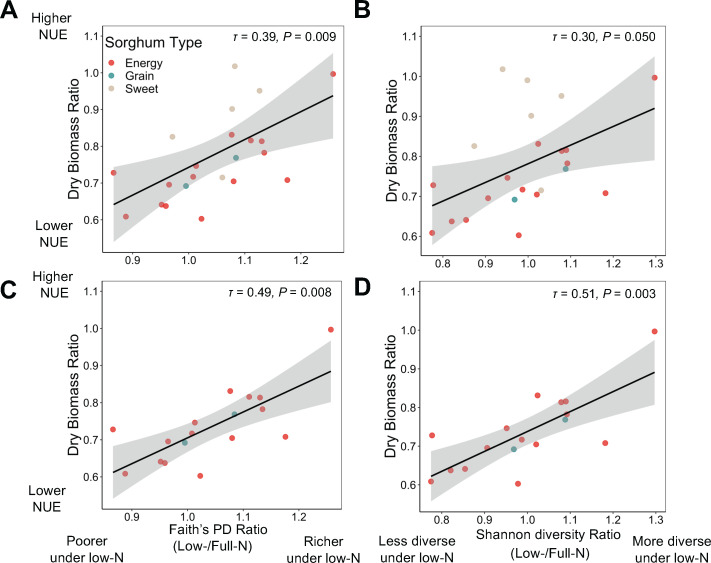
The relationship between bacterial alpha diversity in rhizosphere and sorghum NUE. (**A, C**) The correlation between Faith’s phylogenetic distance and (**B, D**) Shannon diversity ratio (low N/full N) with sorghum NUE derived from dry biomass ratio with (A, B) and without (C, D) sweet sorghum. The coefficient of correlation as Kendall’s τ and *P*-value of each model is denoted on the top right of each plot.

We sought to understand whether the shift in bacterial communities in the rhizosphere and root endosphere due to low N was related to sorghum NUE. First, we correlated the changes in bacterial richness and diversity due to N stress with sorghum NUE expressed as the dry biomass ratio (Fig. 1) across all sorghum genotypes (Fig. 5A and B). The low N-induced changes in the bacterial richness and diversity were derived as the ratios of Faith’s phylogenetic distance and Shannon diversity between low and full N conditions, respectively. We found a significant positive correlation between these ratios and sorghum NUE only in the rhizosphere and not in the root endosphere (Fig. S2), indicating that the genotypes with higher NUE had greater bacterial richness and diversity in the rhizosphere under low N compared with full N. This trend was stronger for the energy and grain sorghum (Fig. 5C and D), but it is important to note that only two grain sorghum genotypes were included in this study.

**Fig 6:**
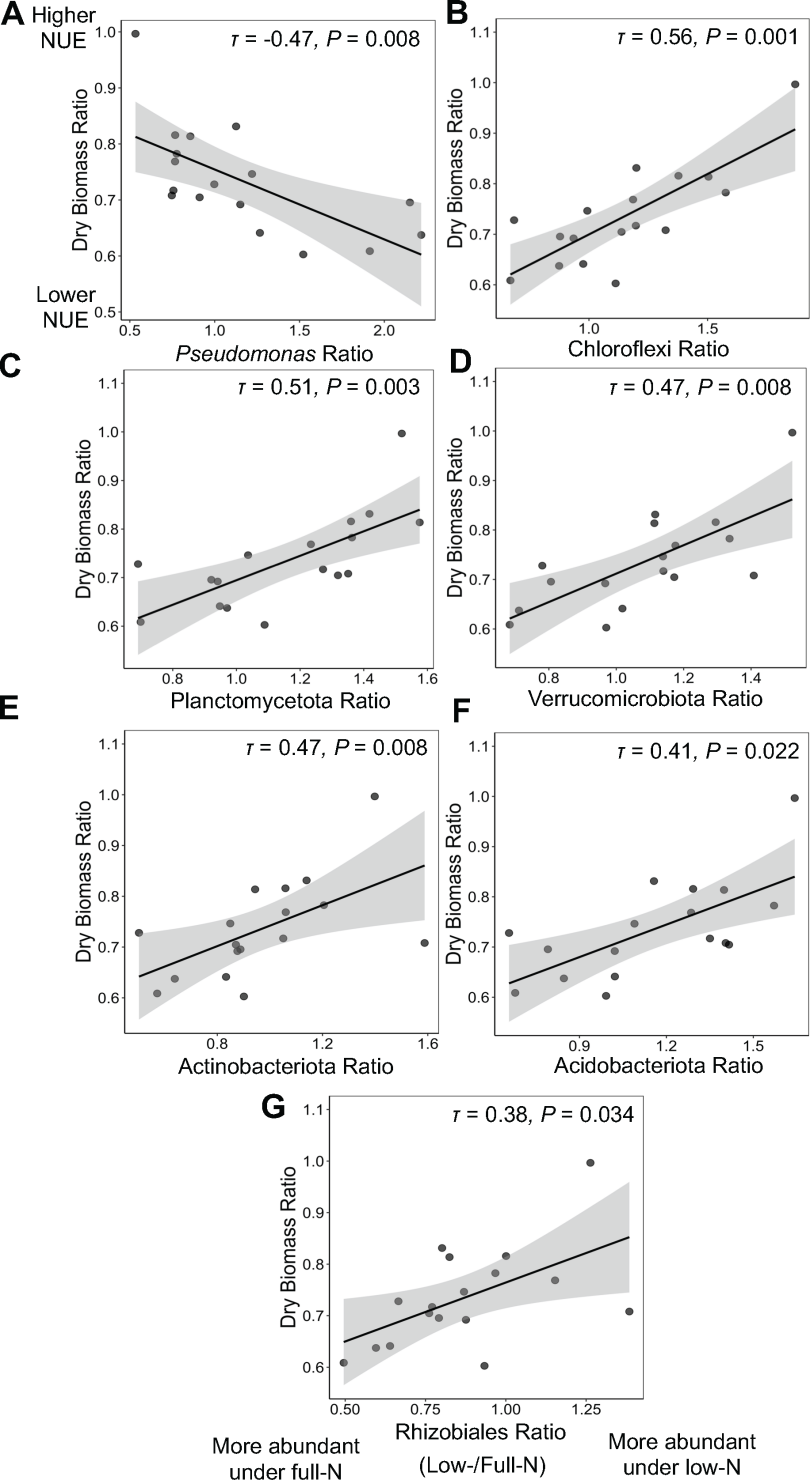
Bacterial taxa in relation to sorghum NUE. Taxa are shown based on two criteria: (i) differential abundance in high versus low nitrogen conditions (LEfSE analysis) and (ii) significant correlations between the differential abundance ratios under low N/full N versus sorghum NUE expressed as the biomass ratios under low N/full N (excluding sweet sorghum) of (**A**) Pseudomonas, (**B**) Chloflexi, (**C**) Planctomycetota, (**D**) Verrucomicrobiota, (**E**) Actinobacteriota, (**F**) Acidobacteriota, and (**G**) Rhizobiales. The coefficient of correlation as Kendall’s τ and *P*-value of each model is denoted on the top right of each plot.

We then investigated the effect of sorghum NUE on individual bacterial taxa using correlation analysis. We selected the microbial taxa across all taxonomic ranks that were responsive to low N as identified by LEfSE and correlated their relative abundance ratio between low and full N with sorghum NUE. We found that the abundance ratio of *Pseudomonas*, the most abundant bacterial genus in the rhizosphere, was negatively correlated with NUE across the energy and grain sorghum type, but not for sweet sorghum (Fig. 6A). In other words, *Pseudomonas* was more abundant in the rhizosphere of the energy and grain sorghum genotypes with lower NUE while it was less abundant in the rhizosphere of the genotypes with higher NUE under low N. Since *Pseudomonas* comprised an average of 27% of the rhizosphere bacterial community of energy and grain sorghum, lower abundance of this genus in the genotypes with higher NUE under low N may be linked to the elevated alpha diversity in the rhizosphere of these sorghum genotypes, and *vice versa* for the low NUE lines. Additionally, the sweet sorghum genotypes had a lower abundance of *Pseudomonas* (~20%) in the rhizosphere than the other two sorghum types, which is in accord with the higher bacterial richness and diversity observed in sweet sorghum’s rhizosphere. The sweet sorghum genotypes tested also had, on average, higher NUE than the grain or energy types. Besides *Pseudomonas*, we identified other bacterial taxa that were differentially enriched or depleted in the rhizosphere of the genotypes with higher or lower NUE. An increased abundance of the phyla of Chloroflexi, Planctomycetota, Verrucomicrobiota, Actinobacteriota, Acidobacteriota, and the order Rhizobiales under low N was observed in the genotypes with higher NUE (Fig. 6B through G).

### Sorghum root metabolite composition is affected by N availability and sorghum NUE

**Fig 7:**
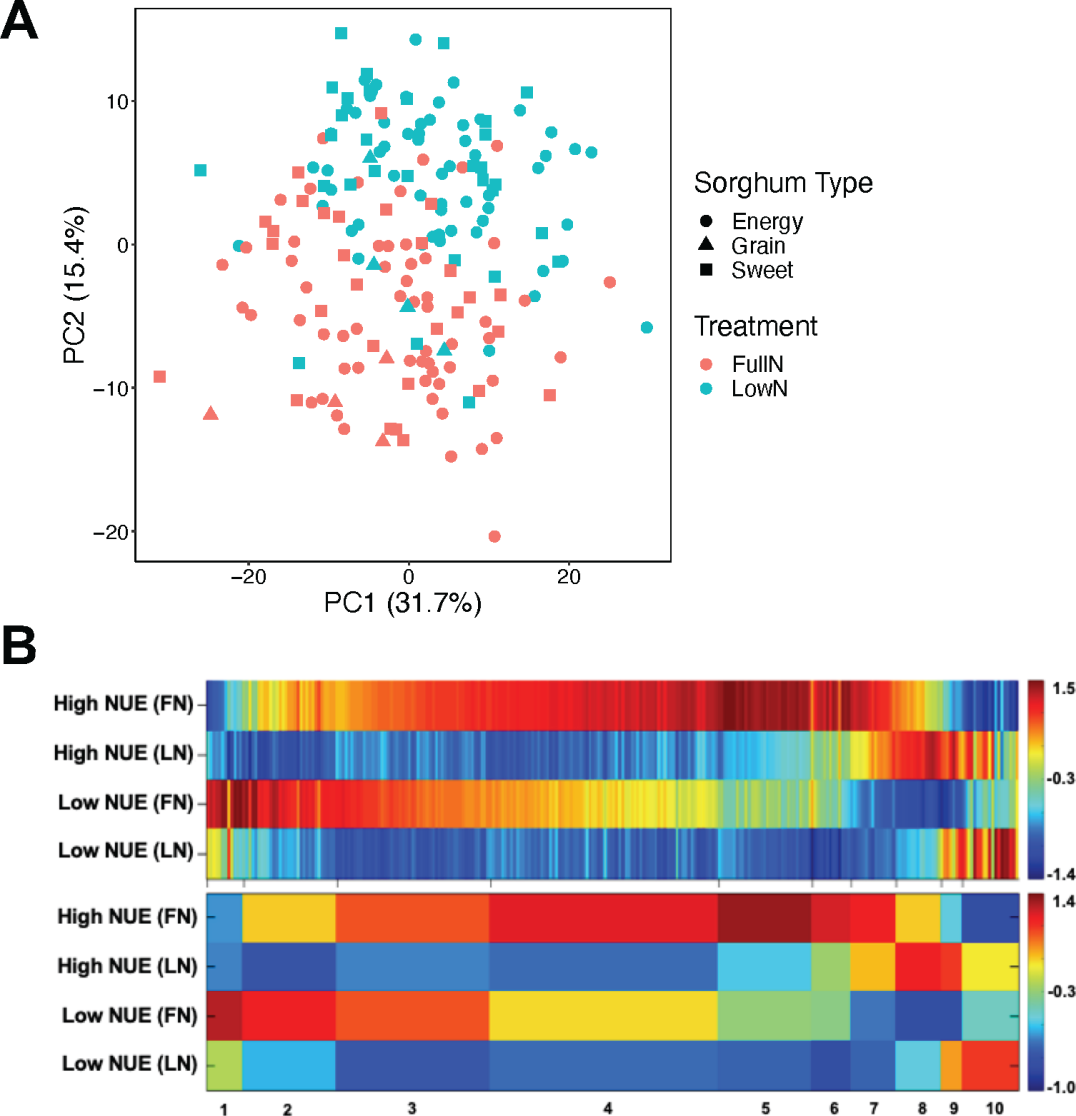
Root metabolomic profiles of sorghum. (**A**) PCA depicting the effects of N treatment and sorghum type on root metabolite composition. (**B**) One-dimensional self-organizing map (1D-SOM) clustering and cluster assignment of sorghum root metabolites. The upper heatmap illustrates the normalized intensity of metabolite features that were significantly affected by N treatment and sorghum NUE as identified by the Kruskal–Wallis test. These metabolites were assigned to 10 clusters in the lower heatmap based on their *z*-scores for the high NUE (NUE > 0.77) and low NUE (NUE < 0.77) lines under each N treatment. The width of each cluster is proportional to the number of metabolite features assigned to the cluster. The color of each cluster represents the mean values of the cluster.

To determine whether low N and sorghum type affected root metabolites, untargeted gas chromatography–mass spectrometry (GC-MS) was deployed to characterize metabolite profiles. The metabolomic profiling was performed on 19 sorghum genotypes (Table S1) with five replicates for each genotype and treatment combination. A total of 429 metabolite features were detected, and annotations were assigned to 77. Principal component analysis (PCA) was used to visualize the variation across samples and showed that the root metabolite profile of the N-stressed plants was clearly different from those that were grown in the full N segments of the field (Fig. 7A). PERMANOVA confirmed that the low N significantly affected the root metabolite profile (*P* < 0.001). While the root metabolite profile was found to differ significantly across the three sorghum types (*P* = 0.037), the difference was not significant in the *post hoc* pairwise comparisons.

The Kruskal–Wallis test was used to identify 270 metabolite features that were differentially affected by the N treatment and sorghum NUE. The 270 metabolite features were then visualized based on their normalized intensity patterns using 1D-SOM (Fig. 7B, upper panel; Data set S1). The metabolite features with similar intensity patterns were grouped into 10 clusters based on their *z*-scores for the high and low NUE lines under each N treatment (Fig. 7B, lower panel; Data set S1). The analysis revealed that 189 metabolite features decreased in intensity under N stress (clusters 2–5). The majority of the metabolite features that decreased under low N were amino acids (asparagine, glycine, leucine, lysine, phenylalanine, serine, valine, and threonine) and organic acids (citric acid, succinic acid, lactic acid, etc.). Conversely, clusters 8–10 showcased metabolite features that were enriched under low N, with cluster 10 highlighting metabolite features that exhibited higher abundance in the low NUE lines (ethanolamine, phytol, etc.) (Fig. 7B; Data set S1). Strikingly, clusters 1 and 7 clearly distinguished the metabolites that were differentially enriched in the low and high NUE lines, respectively. The metabolites that were enriched in low NUE lines included shikimic acid and myo-inositol while the metabolites enriched in high NUE lines included galactinol and tyrosine (Fig. 7B; Data set S1).

### Sorghum root metabolites shape the rhizosphere bacterial community

**Fig 8:**
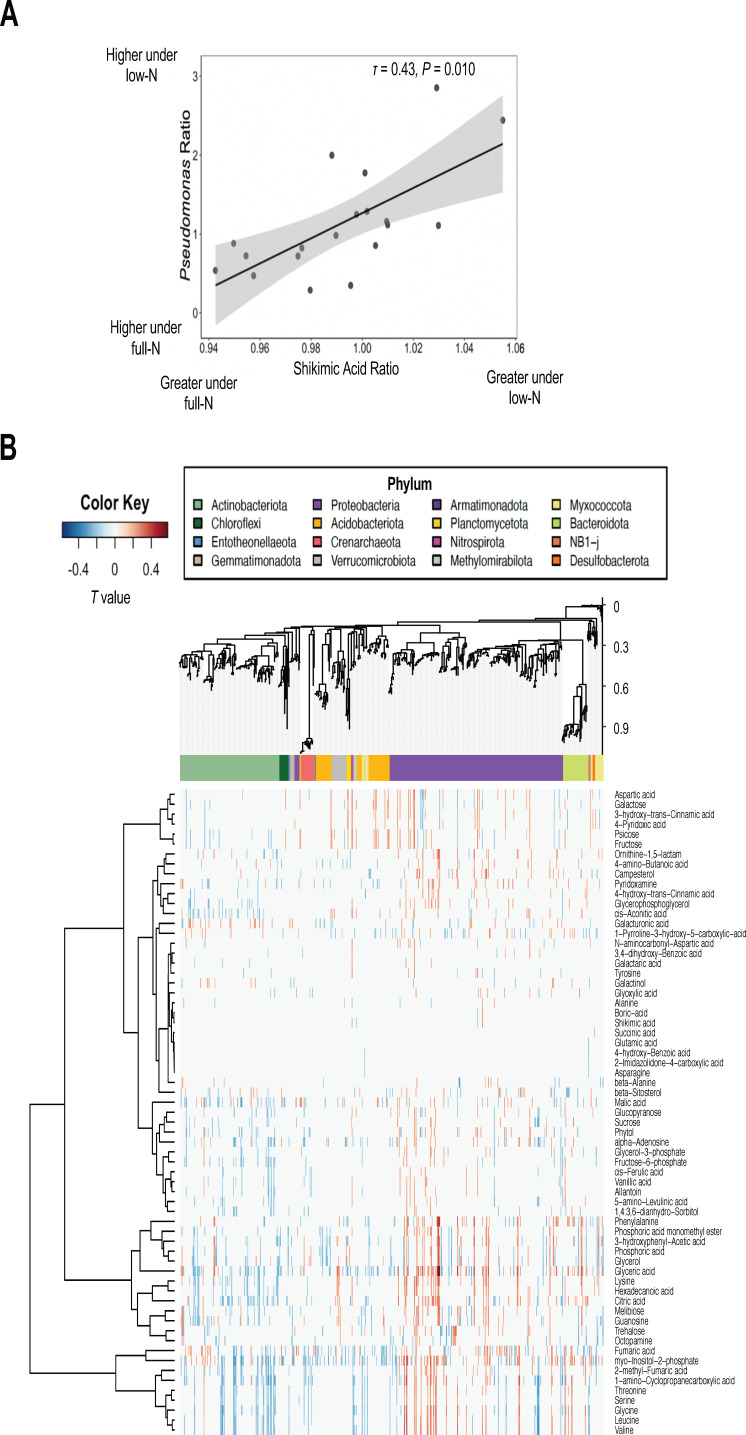
Relationship between root metabolites and rhizosphere bacteria. (**A**) Correlation between *Pseudomonas* ratio (low N/full N) and shikimic acid ratio (low N/full N). (**B**) Heatmap showing correlated metabolites (row) and ASVs (column) identified by GSCCA. The ASVs are arranged based on the position of the ASVs in the phylogenetic tree on the top. The scale bar next to the tree indicates the evolutionary distance of the ASVs. The color bar next to the tree indicates the phylar information of the ASVs.

To determine whether the shifts in the rhizosphere bacterial community were induced by sorghum root metabolites, group sparse canonical correlation analysis (GSCCA) was performed to identify the correlation structure between metabolites and amplicon sequence variants (ASVs). Among the features selected by GSCCA, we identified several metabolites that were correlated with *Pseudomonas* ASVs in the rhizosphere. Despite the fact that 316 ASVs were assigned to *Pseudomonas,* most *Pseudomonas* reads (~91%) were mapped to 10 ASVs, and 8 out of these 10 ASVs were enriched under low N conditions. Interestingly, we found that trehalose was positively correlated with the dominant *Pseudomonas* ASVs (ASV2, 3, 9, 10, and 16; τ ~0.27) that were specifically enriched under low N. We then consolidated all the *Pseudomonas* ASVs and analyzed the correlation between *Pseudomonas* and root metabolites. We found that the ratio of *Pseudomonas* abundance between low and full N exhibited a significant positive correlation with the ratio of shikimic acid between low and full N for all sorghum genotypes tested (Fig. 8A).

In addition to *Pseudomonas*, GSCCA identified a number of ASV and metabolite pairs that were correlated (Fig. 8B; Data set S2). For instance, positive correlations were detected between various amino acids and organic acids with bacterial families that are known for their plant growth-promoting abilities, including *Rhizobiaceae*, *Oxalobacteraceae*, *Comamonadaceae*, and *Burkholderiaceae* (Fig. 8B; Data set S2). Phytol, which was enriched in the low NUE lines under N deficiency, covaried with several members of Actinobacteria and Proteobacteria, but the impact was genus-specific. For example, phytol was positively correlated with *Streptomyces* while negatively correlated with Gaiellales, *Mycobacterium*, *Aeromicrobium*, *Nocardioides*, and *Solirubrobacter* even though they are all members of Actinobacteria (Fig. 8B; Data set S2).

## DISCUSSION

The communication between soil microbial communities and plant root metabolites and exudates is complex and varies depending on plant species, environmental conditions, and plant developmental stage as well as many soil physicochemical factors. It is well known that root metabolites such as nodulation factors stimulate the beneficial interaction between nitrogen fixing legumes and rhizobia under conditions of low N supply. Under conditions of biotic stress, the idea of a “cry for help” has been discussed as a way in which plants deploy metabolite signals to recruit beneficial microbes to help alleviate both abiotic and biotic stresses (24–27). While much of the research enumerating examples of the “cry for help” hypothesis has measured root exudates, root metabolites also provide an important factor in shaping microbial communities (28–30).

Improving NUE in cereal crops is of critical importance to agriculture worldwide. Conventional breeding and genetic engineering are the main approaches currently used to enhance crop NUE (31). The yield enhancement resulting from agronomic approaches where increasing amounts of N fertilizer are used may no longer be a feasible approach to improve crop yield because of the environmental externalities caused by fertilizer usage. Breeding for increased NUE has also been challenging because it has been difficult to identify genes responsible for NUE due to the significant variability in field testing approaches and the likelihood that this is a quantitative trait (31). New methods to improve crop NUE by manipulating plant microbiomes may be a solution that provides a more sustainable alternative since new evidence shows that crop NUE may in part be associated with the plant belowground microbiome (32).

### NUE characteristics in sorghum

The genetic variability of sorghum contributes to the significant variation in the NUE across different sorghum genotypes (17). In this study, we compared the growth of 24 diverse sorghum lines under two N regimes and identified the genotypes that exhibited higher NUE when N was limited. Consistent with other studies (18, 33), we found that sweet sorghum genotypes generally have higher NUE compared with energy and grain sorghum. PI 297155 was one of the genotypes with the highest NUE identified in this study, but it was also determined to have the lowest NUE in our previous greenhouse study (33). This discrepancy in sorghum NUE between greenhouse and field conditions is not surprising because crop NUE could vary with many factors including N fertilization rates (34), soil characteristics (e.g., pH and texture) (35), and the composition of the soil microbiome, highlighting the importance of selecting for crop NUE in different environments. Overall, our study highlights the large degree of NUE variability across diverse sorghum genotypes and provides insight on the potential candidates to be used in the breeding of high NUE sorghum lines.

### Role of microbiome in influencing sorghum NUE

Microbial richness and diversity are frequently demonstrated to be positively correlated with plant vigor (32, 36–38). Yet, our knowledge of the mechanisms underpinning the positive effects of microbial diversity on plant health is limited to the perspective that higher microbial diversity helps buffer against the perturbation of certain microbial species that are usually harmful to plants (36). Our finding that increased microbial richness and diversity in the rhizosphere are positively correlated with plant performance under low N conditions provides evidence that increased bacterial diversity may contribute to mitigating sorghum N stress. This correlation is also indicative of the large variation in responses to low N among the range of sorghum lines studied here that differ in NUE. In agreement with our result, greater bacterial diversity has also been found in the root endosphere of *indica* rice varieties, which exhibit superior NUE compared with the *japonica* lines (32). In the rice study, the increased bacterial diversity was attributed to the recruitment of a large proportion of N cycle-related bacteria in *indica* varieties, which may have enhanced the plant N availability. In another study on tree species when N became limited, plant root metabolites were shown to stimulate microbial decomposition of soil organic matter to increase N mineralization for plant uptake (39, 40). Our findings and those of others suggest that high NUE in plants may in part be linked to the ability to modulate the belowground microbial communities to ultimately improve N uptake and availability. The positive correlation between microbial diversity and plant NUE may be due to enhanced complementary interactions (41) and improved overall metabolic capabilities of rhizosphere microbial communities under low N. This hypothesis warrants further testing given the importance of increasing NUE in modern agroecosystems. A functional consequence of the increased diversity may be to boost soil organic matter decomposition and other aspects of N cycling with positive impacts on the overall productivity of the whole plant–microbe holobiont.

We further revealed that the increased bacterial diversity and richness in the rhizosphere of high NUE genotypes under low N were associated with the decreased abundance of *Pseudomonas* and increased abundance of members of Chloroflexi, Planctomycetota, Verrucomicrobiota, Actinobacteriota, Acidobacteriota, and *Rhizobiales. Pseudomonas* was the most abundant genus identified in the rhizosphere in our study. Strikingly, *Pseudomonas* proliferation particularly during the early host vegetative stage has also been documented in various independent studies on sorghum (8, 42, 43) and maize (44). Although the cause of *Pseudomonas* proliferation is unclear, *Pseudomonas* is a copiotroph specialized in utilizing plant root metabolites (42). Therefore, we hypothesize that the lower abundance of *Pseudomonas* in the high NUE genotypes may be driven by reduced access to labile carbon from plant roots in response to N stress, which facilitated the enrichment of the oligotrophic phyla, Chloflexi, Planctomycetota, Verrucomicrobiota, and Acidobacteriota (45). The enrichment of the order *Rhizobiales* in the rhizosphere may also contribute to plant N uptake as this order contains many N-fixing genera (46). The association between the relative abundance of these taxa and host NUE was stronger in energy and grain, but not in sweet sorghum genotypes that exhibited greater NUE. This suggested that the effect of rhizosphere bacterial communities on sorghum NUE may be related to the sorghum types and the degree of NUE in various genotypes.

### Plant modulation of root metabolites under low N

The availability of N was the primary factor impacting the root metabolite composition in our study, with a notable decrease in many amino acids in N-stressed roots. This finding is in line with previous studies (8, 47). The reduction in amino acids, particularly phenylalanine, may explain the decrease observed in several hydroxycinnamates and hydroxybenzoates derived from shikimic and phenylpropanoid pathways. Many of these compounds (vanillic acid, *p*-benzoic acid, ferulic acid, etc.) are integral constituents of the cell wall or involved in plant defense against pathogens (48). Therefore, the reduction in these compounds under low N may have accounted for the increase in bacterial richness measured in the rhizosphere and root endosphere by lowering the barriers to colonization.

We also demonstrated that certain root metabolite features were differentially enriched or depleted in sorghum lines with different NUE, suggesting that there may be associations between root metabolism and sorghum NUE. For instance, we found that galactinol was differentially enriched in the root of high NUE lines regardless of the N treatment. While galactinol has not been demonstrated to directly link to plant NUE, it has been found to be involved in priming a plant’s defense system against pathogens (49, 50) and enhancing plant tolerance to various abiotic stresses including drought (51), salt (52), and temperature stress (53). The accumulation of galactinol can effectively protect plant cells from damage induced by reactive oxygen species that accumulate under stress conditions (54). On the other hand, phytol accumulation was associated with low NUE lines under N stress. Phytol is a diterpene constituent of chlorophyll, and the accumulation of phytol is often associated with chlorophyll degradation induced by stresses such as N deprivation (55, 56). Therefore, greater phytol accumulation in roots in the low NUE lines further shows that N stress was more severe in these plants than in high NUE genotypes. In addition, both the work by Sheflin et al. (8) and our study found that the concentration of shikimic acid in the sorghum roots was correlated to the differential abundance of *Pseudomonas* in the rhizosphere, consistent with findings that *Pseudomonas* was more abundant in low N fields and could be enriched *in vitro* by shikimic acid addition (42). Shikimic acid is a precursor of aromatic amino acids including phenylalanine, which is required for the synthesis of a plant defense hormone, salicylic acid. Although root salicylic acid was not measured, our results provide further evidence that the differential abundance of *Pseudomonas* in sorghum rhizosphere may be attributed to the varying defense or stress responses in the sorghum lines that differed in NUE under low N conditions (8). Trehalose has been shown to have integral roles in alleviating plant abiotic stresses such as drought and salinity (57). While little is known about the role of trehalose in N stress, in our study, it was related to reduced microbial diversity and lower NUE in sorghum. In contrast, under nitrogen deficiency, an association between cereal species and tolerance to low nitrogen conditions was linked to trehalose (58), but no comparisons were done between genotypes of the same species as in our study. Taken together, our findings highlight the potential crosstalk between host root metabolites and the rhizosphere bacterial communities that may be important for plant performance under low N conditions.

### Conclusion

The objectives of this study were to more fully elucidate how sorghum genotype, root-associated bacteria, and root metabolites may be related to nitrogen use efficiency in sorghum. The findings from our study demonstrate that there are associations between the sorghum root metabolome and rhizosphere bacterial communities that are associated with sorghum NUE across a range of genetically diverse genotypes. We found that sorghum bacterial communities were impacted by the sorghum type (grain, sweet, and energy). N stress was also an important factor that led to the change in the composition of bacterial communities in the rhizosphere and root endosphere. Although the shifts in root metabolite composition were mainly driven by N availability, the sorghum type did influence root metabolite composition. Sorghum NUE was positively correlated with the richness and diversity of the bacterial community in the rhizosphere, which was linked to the differential abundance of the dominant *Pseudomonas* genus. We also showed that the changes in root metabolites due to N stress were correlated with the shifts in the rhizosphere bacterial community composition. Taken together, our results highlight the importance of considering the vast sorghum genetic variation in both plant responses to changes in soil fertility, which led to certain fingerprints of root metabolome and root-associated bacterial community composition. The variation in the metabolome and microbiome composition was also related to the differences in sorghum NUE. Future experiments will be needed to test whether specific microbes and metabolites identified in this study cause changes in sorghum NUE.

## MATERIALS AND METHODS

### Field description and experimental design

The field used in this study was located in central Nebraska, USA, at GPS coordinates 41.201041–97.944750. Pre-season sampling was done in 16 evenly spaced areas throughout the field in the top 30.5 cm and in the 30.5–91.4 cm region of the soil profile. The soil nitrate levels were on average 3.4 ± 2.2 ppm in the top 30.5 cm of the soil and 6.5 ± 3.3 ppm in the 30.5–91.4 cm region of the soil. The field was set up as a split-plot design with eight blocks or replicates that contained two N treatments and 24 sorghum genotypes (Table S1) arranged randomly within each treatment. Plots contained four rows approximately 4.6 meters in length and 76 cm between rows. Eighty-five pounds of granular urea (46-0-0) was added to the full N treatments, and no urea was added to the low N treatments. Seeds were treated with Concep III before planting, and metolachlor and bromoxynil were used just after sorghum germination to help control weeds. Planting was performed on 26 May 2017, and seeds were planted at an interval of 4 inches in a row in the four row plots. Cultivation to remove weeds was done by hand during the season. Sampling was done only on the two inner rows that contained on average 37 plants per the inner two rows of each plot.

### Sample collection and preparation

#### Biomass sampling

Final plant biomass was measured on 9 October 2017 by harvesting a 1-m section of one of the middle rows and then using the area of that section to extrapolate to kilogram/hectare. Stalks and panicles were weighed separately. Four replicate blocks out of the eight were subsampled for dry weight. Plants were weighed, and a subsample of three plants was taken then reweighed, bagged, dried at 80°C, and reweighed to determine the dry weights of the plots. The influence of N treatment and genotype on plant biomass was assessed using linear mixed-effects models through the lmer (59) function in R. Biomass data were log-transformed in the model to correct for skewness. Plots and blocks were included in the model as random effects. For some genotypes, the block effects were found to be insignificant and removed from the model. We estimated a biomass ratio of low N versus full N conditions for each genotype and constructed confidence intervals for the ratio for each genotype based on the linear mixed effects model using the emmeans package (60) in R. This ratio was used to evaluate the NUE of the different sorghum genotypes under limited N conditions. The biomass data of one genotype Chinese Amber could not be measured because most plants had fallen over due to strong winds and weak stems prior to the biomass harvest.

#### Microbiome and root metabolite sampling

Soil between rows, soil within rows, rhizosphere, root, and leaf samples were collected from the field on 18 July 2017. Except for the soil between rows, each sample was collected from two plants located at two different spots within each plot. Soil between rows samples were excavated from the top 30 cm of soil in between plots from different locations of the field. Soil within rows, rhizosphere, and root samples were collected as described previously (61). Leaf samples were collected from the emerging leaf inside the whorl that had not been exposed because they were considered to be absent of leaf epiphytic microbes. The leaf tissue was rinsed in sterile phosphate buffer and cut into small pieces carefully with sterile tools and then stored on ice. In addition to collecting root samples for microbiome analysis, a subset of roots was separated for metabolite profiling after carefully removing the rhizosphere soil by rinsing again in phosphate buffer and gently wiping the roots with a Kimwipe.

All the samples for microbiome and metabolome analyses were brought back to the laboratory on ice and processed as described previously (8, 61). The soil between rows was processed with the same procedure as the soil within rows. In brief, these soil samples were sieved to remove any small roots and debris before freezing at −20°C and then loading into a 96-well plate for DNA extraction. For the leaf samples, a surface sterilization step was omitted because the leaf portion that we collected was still inside the plant and had not emerged from the shoot. Both root tissues and leaf samples were frozen at −80°C and ground in liquid N to homogenize the samples before DNA extraction.

### DNA extraction and sequencing

Following the manufacturer’s protocols, soil and rhizosphere DNA were extracted using the MagAttract PowerSoil DNA KF Kit (Qiagen, Germantown, MD) with the KingFisher Flex System (Thermo Fisher Scientific, Waltham, MA) while the MagMAX Plant DNA Kit (Thermo Fisher Scientific, Waltham, MA) was used for the extraction of leaf and root tissues. The V4 region of 16S rRNA was then amplified with the primers 515F (GTGCCAGCMGCCGCGGTAA)/806R (GGACTACHVGGGTWTCTAAT) followed by sequencing with the Illumina MiSeq platform at the Joint Genome Institute using the protocol described previously (42).

### Non-targeted GC-MS and data analysis

Non-targeted GC-MS was used for root metabolite profiling using the protocol described previously (8). Root metabolite profiling was performed on 19 selected genotypes (excluding Grassl, PI655972, PI505735, BTx623, and Chinese Amber) with five replicates for each genotype and treatment combination. Root tissue was frozen at −80 °C and then lyophilized. Lyophilized root tissue was homogenized in 5 mL polypropylene tubes using stainless steel beads and the Bullet Blender Storm 5 homogenizer (Next Advance, Averill Park, NY). A 20-mg portion of the lyophilized and homogenized tissue was weighed into a 2-mL glass vial, and biphasic extraction was performed by adding 1 mL of methyl-tert-butyl-ether (MTBE) solution containing 6:3:1 MTBE:methanol:water (vol/vol/vol), vortexing at 4°C for 60 min, followed by centrifugation for 15 min at 3,500 rpm. Three hundred fifty microliter of water was added to the extract and vortexed for 30 min at 4°C, followed by centrifugation at 2,750 rpm at 4°C for 15 min. The aqueous layer was transferred to an Ambion filter cartridge (Thermo Fisher Scientific, USA) and was centrifuged briefly to pass the aqueous extract through the column. Two hundred microliter of the filtered aqueous layer was dried down completely under N_2_ (g). The dried samples were re-suspended in 50 µL of pyridine containing 25 mg/mL of methoxyamine hydrochloride (Sigma), incubated at 60°C for 45 min, vigorously vortexed for 30 s, sonicated for 10 min, and incubated for an additional 45 min at 60°C. Next, samples were cooled to room temperature and briefly centrifuged. Then, 50 µL of N-methyl-N-trimethylsilyltrifluoroacetamide with 1% trimethylchlorosilane (MSTFA + 1% TMCS, Thermo Fisher Scientific) was added; samples were vigorously vortexed for 30 s and then incubated at 60°C for 30 min. Metabolites were separated and detected using a Trace 1310 GC coupled to an ISQ mass spectrometer (Thermo Fisher Scientific). Samples (1 µL) were injected at a 10:1 split ratio onto a 30-m TG-5MS column (0.25 mm i.d., 0.25 µm film thickness; Thermo Fisher Scientific) with a 1.2-mL/min helium gas flow rate. The gas chromatography inlet was held at 285°C. The oven program started at 80°C for 30 s, followed by a ramp of 15°C/min to 330°C and an 8 min hold. Masses between 50 and 650 *m*/*z* were scanned at 5 scans/sec under electron impact ionization. The transfer line and ion source were held at 300 and 260°C, respectively.

GC-MS data were processed using the R statistical environment as described previously (62). Briefly, the processing steps follow: (i) XCMS software defined a matrix of molecular features (63), (ii) samples were normalized to total ion current, (iii) RAMClust package for R clustered co-varying and co-eluting features into spectra (64), and (iv) RAMSearch software (65) allowed annotation by searching spectra against internal and external databases. Databases used for annotations included golm (http://gmd.mpimp-golm.mpg.de/) and NISTv14 (http://www.nist.gov). The log2-transformed and pareto-scaled metabolite data (annotated metabolites and unknowns) were then subjected to principal component analysis using prcomp (66) function in R. The effects of N treatment and sorghum type were assessed using their Bray–Curtis distance through adonis (67) function in R, and the split-plot design was considered using the parameter “strata.” Subsequently, the metabolite data were subjected to clustering using 1D-SOM through MarVis software (version 2.0, http://marvis.gobics.de) (68, 69). Only the metabolites that were differentially affected by N treatment and NUE as identified by the Kruskal–Wallis test were used in the 1D-SOM analysis (69). The metabolites were normalized based on their *z*-scores (subtracting the mean then dividing by the standard deviation) and aggregated into 10 clusters by their mean values.

### 16S amplicon data analysis

The analysis of 16S amplicon data was done using UPARSE (70) and QIIME 2 (71), and R (66) was used for figure generation. UPARSE was used to merge the paired-end reads, remove the primers, filter out the reads with expected error scores below one, remove chimeras, and generate read clusters at 100% similarity. The resulting table containing the read count of the ASVs was then subjected to downstream analyses using QIIME 2. Taxonomy was assigned to each ASV using a q2-feature-classifier (72) pre-trained with the SILVA database (73). Reads assigned to chloroplast and mitochondria were removed, constituting an average of 88%, 38%, 1.5%, 1.7%, and 1.2% of the total reads in leaf, root, rhizosphere, soil within rows, and soil within rows, respectively (Table S3). Rarefaction curves were generated for each sample type after the chloroplast and mitochondria sequences were removed and prior to the alpha and beta diversity analyses to ensure that a sufficient sampling depth was achieved. Sampling depths of 84,009, 57,118, 80,141, 15,496, and 500 were used for soil between rows, soil within rows, rhizosphere, root endosphere, and leaf, respectively. For the alpha diversity analyses, the Shannon diversity index and Faith’s phylogenetic distance were used to assess the diversity and richness of the microbiome, respectively. The difference in alpha diversity indices for each combination of N treatment and sample type was tested using ANOVA in R followed by *post hoc* Tukey–HSD pairwise comparisons. For the beta diversity analyses, PCoA and CAP were performed on Bray–Curtis distance matrices using pcoa (74) and capscale (67) functions in R. The changes in microbiome composition due to N treatment, sample type, sorghum genotype, and sorghum type were evaluated using PERMANOVA with 999 permutations using adonis function (67) in R, and the split-plot design was considered in the model using the parameter “strata.” Pairwise comparisons across the different sorghum types were then conducted using PERMANOVA, and the *P*-values were adjusted using Benjamini–Hochberg false discovery (FDR) correction. The bacterial taxa differentially affected by N treatment and sorghum NUE were identified using linear discriminant analysis effect size analysis based on the relative abundance at http://huttenhower.sph.harvard.edu/lefse/ (75). A non-parametric measure of correlation, Kendall’s rank correlation, was employed to evaluate the association between NUE and the alpha diversity ratios (Shannon diversity index and Faith’s phylogenetic distance) for each sorghum genotype using the cor.test (66) function in R. These alpha diversity ratios were calculated by dividing the mean values under low N by those under high N for each genotype. To determine the relationships between relative abundance ratios (low N/high N) of bacterial taxa significantly impacted by N treatment, as identified by LEfSE, and sorghum NUE based on biomass ratios, we first identified the taxa exhibiting differential abundance in high versus low nitrogen conditions (based on LEFSE). We then only show the taxa that had significant correlations between the abundance ratios and NUE (Fig. 6).

### Correlation between metabolites and rhizosphere microbiome

GSCCA was used to identify the correlations between root metabolites and rhizosphere ASVs (76). Only the samples that were profiled for both root metabolite and microbiome were included in this analysis. The ASVs present in less than 95% of the samples for any combination of sorghum type and genotype in both low and full N treatments were excluded from the analysis. Metabolites that were unannotated were also excluded from GSCCA. As a result, a total of 690 ASVs and 77 metabolites were included in the GSCCA. The filtered ASV data were subjected to analysis of composition of microbiomes transformation (77) while the metabolite data were log-transformed. Next, we grouped the ASVs and metabolites based on their phylum and superclass, respectively. Superclass is the second-level chemical taxonomy assigned to biochemicals according to their structural features (The Metabolomics Workbench, https://www.metabolomicsworkbench.org/). We obtained the between-group and within-group correlations, and the groups with high between-group correlations were merged until the within-group correlations were higher than the between-group correlations. We also merged the superclass or phylum singletons with the corresponding most correlated groups. After merging, we performed GSCCA based on the pooled covariance with respect to the full and low N groups to select groups and features that contribute to the canonical correlations between metabolites and ASVs, followed by a test with 10,000 permutations to determine if the canonical correlations were significantly different from zero. To account for the imbalance of sample sizes in different genotypes, we used stratified cross-validation when searching for the best tuning parameters.

As *Pseudomonas* was identified as the most prevalent genus in the sorghum rhizosphere in this study and exhibited higher abundance in the low NUE lines, we conducted Kendall’s rank correlation to evaluate the association between the ratios of *Pseudomonas* genus and shikimic acid, which was enriched in the root of low NUE lines. Both of these ratios were derived by dividing the mean values under low N by those under high N.

## Data Availability

The 16S rRNA sequences used in this study are available at the NCBI repository under the BioProject number PRJNA830495. GC-MS data have been deposited to the MassIVE database (DOI: 10.25345/C58S4K05W) with the identifier MSV000092783. The complete data set can be accessed here: https://massive.ucsd.edu/ProteoSAFe/static/massive.jsp using MSV000092783 as the search term. The scripts for the analyses are available at https://github.com/yenning-chai/CentralCityManuscript.

## SUPPLEMENTAL MATERIAL

Data Set S1 msystems.01190-23-s0001.xlsx GC-MS metabolite data.

Data Set S2 msystems.01190-23-s0002.xlsx Correlated metabolite and ASV pairs identified by group sparse canonical correlation analysis.

Fig. S1 msystems.01190-23-s0003.tiff Biomass data for each genotype.

Fig. S2 msystems.01190-23-s0004.tiff Relationship between bacterial alpha diversities in the root endosphere and NUE.

Supplemental material msystems.01190-23-s0005.pdf Tables S1 to S3 and legends for Fig. S1 and S2.

ASM does not own the copyrights to Supplemental Material that may be linked to, or accessed through, an article. The authors have granted ASM a non-exclusive, world-wide license to publish the Supplemental Material files. Please contact the corresponding author directly for reuse.
