# Supplementary figures and images for: Root-associated bacterial communities and root metabolite composition are linked to nitrogen use efficiency in sorghum
Source: mSystems. 2023 Dec 22;9(1):e01190-23. doi: 10.1128/msystems.01190-23 (PMC10804983; doi:10.1128/msystems.01190-23)

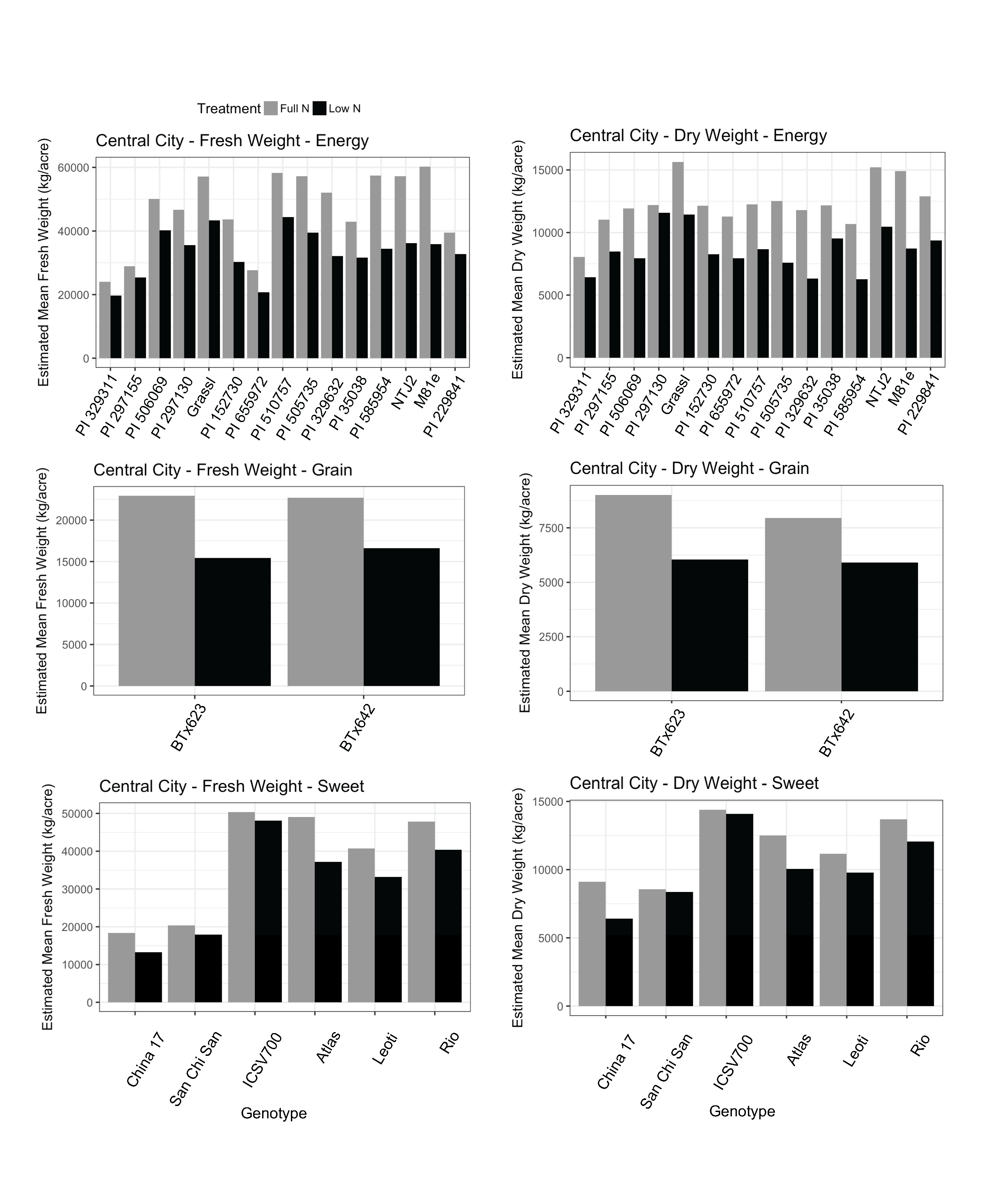

Supplement: Fig. S1 — Biomass data for each genotype. [file msystems.01190-23-s0003.tiff]
