# Supplementary material for: Root-associated bacterial communities and root metabolite composition are linked to nitrogen use efficiency in sorghum
Source: mSystems. 2023 Dec 22;9(1):e01190-23. doi: 10.1128/msystems.01190-23 (PMC10804983; doi:10.1128/msystems.01190-23)
Supplement: Supplemental material — Tables S1 to S3 and legends for Fig. S1 and S2. [file msystems.01190-23-s0005.pdf]

Table S1. Sorghum genotypes used in this study and genotypes from which metabolite data were collected. The collection of samples for microbiome characterization was performed on all genotypes. Biomass data was measured for all genotypes except Chinese Amber due to severe lodging prior to the harvest.

Genotype	Type	Race	Root Metabolite
PI 329311	Energy	Durra	Yes
PI 297155		Kafir	Yes
PI 506069		Guinea/bicolor	Yes
PI 297130		Caudatum	Yes
Grassl		Caudatum	No
PI 152730		Caudatum/bicolor	Yes
PI 655972		Kafir	No
PI 510757		Durra	Yes
PI 505735		Caudatum	No
PI 329632		Durra	Yes
PI 35038		Caudatum	Yes
PI 585954		Guinea	Yes
NTJ2		Durra	Yes
M81e		Caudatum/durra	Yes
PI 229841		Kafir	Yes
BTx623	Grain	Kafir	No
Btx642		Caudatum	Yes
China 17	Sweet	Bicolor	Yes
San Chi San		Bicolor	Yes
ICSV700		Bicolor	Yes
Atlas		Kafir	Yes
Leoti		Kafir/bicolor	Yes
Chinese Amber		Bicolor	No
Rio		Durra/caudatum	Yes

Table S2. Bacterial community composition in the soil, rhizosphere, root, and leaf endosphere were compared across the three different sorghum types based on their Bray-Curtis distance using PERMANOVA with 999 permutation. The *p*-values shown have been adjusted using Benjamini-Hochberg FDR correction.

**Sample type**	**Groups compared**	**Sample size**	**pseudo-F**	***p*-value**
Soil	Energy vs. Grain	99	4.840	0.002
	Energy vs. Sweet	126	1.968	0.026
	Grain vs. Sweet	57	3.467	0.002
Rhizosphere	Energy vs. Grain	196	2.835	0.017
	Energy vs. Sweet	253	1.757	0.074
	Grain vs. Sweet	101	2.551	0.017
Root	Energy vs. Grain	227	1.853	0.002
	Energy vs. Sweet	292	1.591	0.008
	Grain vs. Sweet	123	2.052	0.002
Leaf	Energy vs. Grain	135	2.807	0.002
	Energy vs. Sweet	170	2.969	0.003
	Grain vs. Sweet	71	4.649	0.002

Table S3. The proportion of host reads (mitochondria and chloroplast) present in each sample types.

	Total reads before removing host reads			Host reads		
Sample type	Mean	Median	Range	Mean (%)	Median (%)	Range (%)
Soil between rows	100091.87	106558	645 - 197641	1.20	1.59	0.82 - 3.75
Soil within rows	99376.52	100070	101 - 272317	1.65	1.52	0.73 - 7.67
Rhizosphere	108370.11	101554	1 - 590943	1.50	1.47	0 - 4.96
Root	80437.06	80264	7 - 259343	38.02	37.79	0 - 93.59
Leaf	107086.23	100766	6 - 327514	88.33	94.87	2.89 - 99.98

Figure S1. Biomass of 23 diverse sorghum genotypes. Fresh (left) and dry weight (right) of each sorghum genotype under full- and low-N.

Figure S2. The relationship between bacterial alpha diversities in root endosphere and sorghum NUE. (A,C) The correlation between Faith's phylogentic distance, (B,D) Shannon diversity ratio (low-N/full-N) with sorghum NUE derived from dry biomass ratio with (top) and without sweet sorghum (bottom). Coefficient of correlation as Kendall's τ and *p*-value of each model is denoted on the top right of each plot.
